# A comparison of brain retraction mechanisms using finite element analysis and the effects of regionally heterogeneous material properties

**DOI:** 10.1007/s10237-023-01806-2

**Published:** 2024-02-15

**Authors:** Emma Griffiths, Jayaratnam Jayamohan, Silvia Budday

**Affiliations:** 1 Department of Mechanical Engineering, Institute of Continuum Mechanics and Biomechanics, Friedrich-Alexander-Universität Erlangen-Nürnberg, 91058 Erlangen, Germany; 2 Department of Pediatric Neurosurgery, John Radcliffe Hospital, Oxford, OX3 9DU UK

**Keywords:** Human brain, Neurosurgery, Finite element method, Material modelling, Regional heterogeneity

## Abstract

Finite element (FE) simulations of the brain undergoing neurosurgical procedures present us with the great opportunity to better investigate, understand, and optimize surgical techniques and equipment. FE models provide access to data such as the stress levels within the brain that would otherwise be inaccessible with the current medical technology. Brain retraction is often a dangerous but necessary part of neurosurgery, and current research focuses on minimizing trauma during the procedure. In this work, we present a simulation-based comparison of different types of retraction mechanisms. We focus on traditional spatulas and tubular retractors. Our results show that tubular retractors result in lower average predicted stresses, especially in the subcortical structures and corpus callosum. Additionally, we show that changing the location of retraction can greatly affect the predicted stress results. As the model predictions highly depend on the material model and parameters used for simulations, we also investigate the importance of using region-specific hyperelastic and viscoelastic material parameters when modelling a three-dimensional human brain during retraction. Our investigations demonstrate how FE simulations in neurosurgical techniques can provide insight to surgeons and medical device manufacturers. They emphasize how further work into this direction could greatly improve the management and prevention of injury during surgery. Additionally, we show the importance of modelling the human brain with region-dependent parameters in order to provide useful predictions for neurosurgical procedures.

## Introduction

Retraction of the brain is often necessary during neurosurgery to access problematic areas of the brain. It traditionally involves manoeuvring brain tissue using a spatula retractor to access deep parts of the brain in order to remove or repair tumours or lesions. Secondary damage is often an unfortunate result of retraction (Andrews and Bringas 1993; Zhong et al. 2003). This is considered damage to the surrounding tissue that was not planned or accounted for initially. Damage to nearby subcortical structures (Iyer and Chaichana 2018), excessive severing of white matter (Raza et al. 2011; Bander et al. 2016), and tissue creep (Kassam et al. 2015) can result in secondary neurological complications resulting in functional impairment of patients.

Using tubular retractors instead of traditional spatulas has been suggested to reduce the amount of secondary brain damage (Okasha et al. 2020; Jamshidi et al. 2020; Shapiro et al. 2020; Eichberg et al. 2020; Mansour et al. 2020). Tubular retractors consist of a cylinder or cone with either a circular or elliptical cross section. These tubes are inserted in the brain tissue and provide a surgical corridor through which the deep parts of the brain can be accessed. These retractors offer two obvious advantages over traditional spatulas: smaller incisions of the brain tissue (reducing primary damage) and an even distribution of pressure on retracted brain tissue compared to the hard edges of spatulas (reducing secondary damage due to shearing forces on the brain) (Zagzoog and Reddy 2020; Evins 2017; Raza et al. 2011).

There are several clinical reports describing the successful use of tubular retractors on tumours and lesions that are intra-axial (tumour or lesions found within the parenchyma that is likely to be near subcortical structures) (Kelly et al. 1988; Raza et al. 2011; Recinos et al. 2011; Bander et al. 2016; Gassie et al. 2018; Mansour et al. 2020; Marenco-Hillembrand et al. 2018, 2020; Echeverry et al. 2020), deep-seated high-grade gliomas (Iyer and Chaichana 2018), and lesions within the ventricles of the brain (Cohen-Gadol 2013; Shoakazemi et al. 2015). Additionally, they have been used for the biopsies of deep-seated tumours (as they allow for larger biopsy samples to be collected thus allowing for better diagnostic suitability) (Jackson et al. 2017; Bander et al. 2018) and aneurysm clippings (Jamshidi et al. 2018; O’Connor et al. 2019; Jamshidi et al. 2020). These types of surgical procedures have a high degree of risk, but are necessary for many patients’ well-being. Thus, taking every measure to reduce secondary neurological deficits is important. There are many accounts in the literature that promote the use of tubular retractors and acknowledge their benefits (Raza et al. 2011; Shapiro et al. 2020; Mansour et al. 2020; Eichberg et al. 2020; Evins 2017).

However, without studies comparing the use of spatula and tubular retractors, one cannot confidently conclude that tubular retractors reduce the incidence of secondary brain injury (Raza et al. 2011; Bander et al. 2016; Eichberg et al. 2020) and further comparative studies are necessary to do so. Making clinical comparison between retraction methods is, however, very difficult to achieve due to several factors such as,

- Variability in patients: Age, gender, and the patient’s current condition can affect brain tissue stiffness. Additionally, there exists anatomical variability in the shape and size of each patient’s brain.
- Variability in surgery: Location, depth and size of lesion/tumour will be different for each patient.
- Variability in surgeons: Techniques, preferences and surgical experience will differ.

FE simulations can provide a means to overcome, and even probe, many of these variabilities. FE simulations also allow for a high risk procedure to be performed with no risk to patients. Additionally, the incidence of secondary brain injury can be difficult to determine (Evins 2017). The resulting impairment to subcortical structures may only show up several days after surgery. FE simulations could provide data on the loading experienced by these subcortical structures during planning in order to minimize the corresponding risk.

FE simulations have previously been used for a variety of neurosurgical applications. Miga et al. (2001) performed a computational study using a linear elastic model computational brain model to simulate a procedure involving retraction and tumour resection so as to improve the patient-to-image registration necessary for surgical image guidance systems. Li et al. (2016) used a hyper-viscoelastic model in conjunction with the extended finite element method (XFEM) to model a similar procedure of a porcine brain based on boundary conditions extracted from *in vivo* experiments. Hansen et al. (2004) included the FE method to enhance haptic feedback of a virtual reality system used to simulate retraction. These systems aim to reduce brain tissue damage by training new surgeons and improve neurosurgical planning. Research has continued in this direction in order to enhance the realism and speed of these virtual reality surgical simulators (Platenik et al. 2002; Fukuhara et al. 2014a, b; Sase et al. 2015). Simulating a retraction surgery, Awasthi et al. (Awasthi et al. 2020) investigated the reaction force and pressure on spatulas using a hyper-viscoelastic heterogeneous canine brain model. They probed how intermittent verse continuous retraction, the number of spatulas used, and the speed of retraction affect these measures. Adachi et al. (2007) simulated the deformation of a patient-specific three-dimensional FE brain model during a retraction surgery using a traditional spatula. Using a porcine brain model, Lamprich and Miga (2003) modelled retraction using FE in order to update preoperative images during surgery.

In order to provide accurate predictions of stress and strain in FE simulated brains, a sufficiently accurate material model of brain tissue is required. However, the brain is a highly complex organ. Brain tissue is extremely soft and compliant and is viscoelastic and/or poro-elastic depending on the time scale of interest and the loading conditions (Budday et al. 2019). In addition, the brain exhibits clear microstructural heterogeneity due to different functional demands in different regions of the brain (Reiter et al. 2021). This results in regionally different macroscopic mechanical properties (Hinrichsen et al. 2023).

Region-specific properties in FE brain models have been used when modelling traumatic head injuries (Viano et al. 2005; Mao et al. 2013; Miller et al. 2016). However, investigation into the importance of this is still in its infancy. In recent investigations, the effects of region-specific hyperelastic parameters by Griffiths et al. (Griffiths et al. 2023) showed that hyperelastic regional heterogeneity produced significantly different results when compared to an homogeneous model, especially in the region of the corpus callosum. This region has been shown to be significantly more compliant than the other regions of the brain (Budday et al. 2019). To the best of our knowledge, the effects of regional heterogeneity of viscoelastic properties for full-scale brain simulations have not been explored.

Creating a model of the brain that can capture the anatomical and material characteristics of the brain under neurosurgical loadings with suitable accuracy is important. It can help us predict how these procedures affect the stress levels within the brain and thus assist in surgical planning. It can also help understand how to improve these procedures and the equipment used in order to prevent or reduce injury (Kyriacou et al. 2002).

To the best of our knowledge, there are no studies comparing different retraction mechanisms utilizing the FE method in conjunction with a brain based on medical images that is segmented into regions with different material properties. Additionally, the effects of retraction on surrounding subcortical structures have not been probed. Both of these investigations would be difficult, if not impossible, to performed with current medical technology. This paper will simulate the retraction of brain tissue using three different types of retractors: (1) traditional spatulas, (2) tubular retractors with an circular cross section and (3) tubular retractors with a elliptical cross section. We compare the effects of each mechanisms on the surrounding cortical and subcortical structures as well as the sensitivity of these methods to the retraction location. Additionally, we investigate the importance of region-specific hyperelastic and viscoelastic parameters. We use a three-dimensional fully segmented brain with a hyper-viscoelastic model, which considers region-specific parameters of four regions of the brain, the cortex, corona radiata, basal ganglia, and corpus callosum (Budday et al. 2017b, a).

## Methods

### Modelling finite viscoelasticity

#### Kinematics

To model the deformation of the brain, we use nonlinear continuum mechanics and consider a deformation mapping $\varvec{\varphi }\,({\textbf{X}},t)$ that maps the undeformed, unloaded configuration with positional vectors ${\textbf{X}}$ at time $t_0$ to the deformed, loaded configuration with position vectors ${\textbf{x}} = \varvec{\varphi }\,({\textbf{X}},t)$ at time *t*. The spectral representation of the deformation gradient, ${\textbf{F}} = d\varvec{\varphi }/d{\textbf{X}} = \nabla \,{\textbf{x}}\,\varvec{\varphi }$, in terms of the eigenvalues $\lambda _\textrm{a}$ is

1 $$\begin{aligned} {\textbf{F}} = \nabla \,{\textbf{x}}\,\varvec{\varphi } = \sum _{\textrm{a}=1}^{3} \lambda _\textrm{a}\, {\textbf{n}}_{\textrm{a}} \otimes {\textbf{N}}_{\textrm{a}}, \end{aligned}$$

where ${\textbf{n}}_{\textrm{a}} = {\textbf{F}}\cdot {\textbf{N}}_{\textrm{a}}$ and ${\textbf{N}}_{\textrm{a}}$ are the eigenvectors in the deformed and undeformed configurations.

We also introduce the spectral representation of the left Cauchy–Green deformation tensor,

2 $$\begin{aligned} {\mathbf{b}} = {\mathbf{F}}\cdot {\mathbf{F}}^{\textrm{t}} = \sum _{\textrm{a}=1}^{3} \lambda _\textrm{a}\, {\mathbf{n}}_{\textrm{a}} \otimes {\mathbf{n}}_{\textrm{a}}. \end{aligned}$$

To model the viscous nature of brain tissue, the deformation gradient is decomposed into an elastic and viscous part,

3 $$\begin{aligned} {\mathbf{F}} = {\mathbf{F}}^{\textrm{e}}_{i} \cdot {\mathbf{F}}^{\textrm{v}}_{i} \quad \forall \quad i = 1,\ldots ,m \,, \end{aligned}$$

where *i* denotes the parallel arrangement of *m* viscoelastic elements (Sidoroff 1974). To characterize the rate of deformation, we introduce the spatial velocity gradient,

4 $$\begin{aligned} {\mathbf{l}} = \nabla _{{\mathbf{X}}}{\mathbf{v}} = \dot{{\mathbf{F}}} \cdot {\mathbf{F}}^{-1} = {\mathbf{l}}^\textrm{e}_{i} + {\mathbf{l}}^\textrm{v}_{i} \end{aligned}$$

which is decomposed into an elastic ${\textbf{l}}^\textrm{e}_{i} = \dot{{\textbf{F}}^\textrm{e}} \cdot ({\textbf{F}}^\textrm{e}_{i})^{-1}$, and a viscous part ${\textbf{l}}^\textrm{v}_{i} = {\textbf{F}}^\textrm{e}_{i} \cdot \dot{{\textbf{F}}^\textrm{v}_{i}} \cdot ({\textbf{F}}^\textrm{v}_{i})^{-1} \cdot ({\textbf{F}}^\textrm{e}_{i})^{-1}$. It proves convenient to introduce the elastic left Cauchy–Green strain tensor for each mode

5 $$\begin{aligned} {\textbf{b}}^{\textrm{e}}_i = {\textbf{F}}^{\textrm{e}}_i \cdot ({\textbf{F}}^\textrm{v}_{i}){}^{\textrm{t}} = \sum _{\textrm{a} =1}^3 (\lambda _{i\,\textrm{a}}^{{\textrm{e}}})^2 \; {\textbf{n}}_{i\,\textrm{a}}^{\textrm{e}} \otimes {\textbf{n}}_{i\,\textrm{a}}^{\textrm{e}} , \end{aligned}$$

with eigenvalues $\lambda _{i\,\textrm{a}}^{{\textrm{e}}}$ and eigenvectors ${\textbf{n}}_{i\,\textrm{a}}^{\textrm{e}}$, which are, in general, not identical to the eigenvectors of the total left Cauchy–Green deformation tensor, ${\textbf{n}}_{i\,\textrm{a}}^{\textrm{e}} \ne {\textbf{n}}_{\textrm{a}}$. The material time derivative of the elastic left Cauchy–Green deformation tensor ${\textbf{b}}^{\textrm{e}}_i$

6 $$\begin{aligned} \dot{{\textbf{b}}}{}^{\textrm{e}}_i = 2\,[\,{\textbf{l}}_i^{\textrm{e}} \cdot {\textbf{b}}^{\textrm{e}}_i\,]^{\textrm{sym}} = 2\,[\,{\textbf{l}} \cdot {\textbf{b}}^{\textrm{e}}_i\,]^{\textrm{sym}} - 2\,[\,{\textbf{l}}^{\textrm{v}}_i \cdot {\textbf{b}}^{\textrm{e}}_i\,]^{\textrm{sym}}, \end{aligned}$$

introduces its Lie-derivative

7 $$\begin{aligned} {\mathscr {L}}_v \, {\textbf{b}}^{\textrm{e}}_i = - 2\,[\, {\textbf{l}}^{\textrm{v}}_i \cdot {\textbf{b}}^{\textrm{e}}_i\, ]^{\textrm{sym}} , \end{aligned}$$

along the velocity field of the material motion.

#### Constitutive modelling

Previously, it has been shown that the Ogden model represents the time-independent, hyperelastic response of the brain tissue under various loading modes (Budday et al. 2017a; Mihai et al. 2015; Miller and Chinzei 2002). The viscoelastic extension of this model has, thereafter, been shown to capture the conditioning and hysteresis effects. Based on previous experimental evidence, we assume an isotropic material response for both the elastic and viscoelastic behaviour (Budday et al. 2019, 2017b).

The viscoelastic free energy function $\psi$ is given as the sum of an equilibrium part $\psi ^{eq}$ given in terms of the total principal stretches $\lambda _a$ and a non-equilibrium term in terms of the sum of the elastic principal stretches $\psi ^{\textrm{neq}} = \sum _{i-1}^{m}\psi _{i}$ for each viscoelastic mode $i,\ldots ,m$.

8 $$\begin{aligned} \psi = \psi ^{\textrm{eq}}+\psi ^{\textrm{neq}}. \end{aligned}$$

Following the standard arguments of thermodynamic, the Kirchhoff stress $\varvec{\tau }$ consists of two terms, the equilibrium term $\varvec{\tau }^{\textrm{eq}}$ and the non-equilibrium term $\varvec{\tau }^{\textrm{neq}}$ which is the sum of the Kirchhoff stress for each viscoelastic mode,

9 $$\begin{aligned} \varvec{\tau } = 2\,\dfrac{\partial \psi }{\partial {\textbf{b}}} \cdot {\textbf{b}}= & {} \varvec{\tau }^{\textrm{eq}} + \varvec{\tau }^{\textrm{neq}} \nonumber \\{} & {} \textrm{ with } \quad \varvec{\tau }^{\textrm{neq}} = \sum _{i=1}^{m}\varvec{\tau }_i. \end{aligned}$$

The equilibrium free energy part is modelled using a one-term Ogden model whereby the strain energy function is split into an isochoric and a volumetric part (Ogden 1972),

10 $$\begin{aligned} \psi ^\textrm{eq} = \psi _{\textrm{iso}}+\psi _{\textrm{vol}}. \end{aligned}$$

The isochoric part is defined in terms of the isochoric principal stretches ${{\bar{\lambda }}}_a=J^{-1/3}\lambda _a$, where *J* denotes the volume ratio $J=\textrm{det} {\textbf{F}}$, and is given by

11 $$\begin{aligned} \psi _{\textrm{iso}} = 2\,\mu _{\infty }/\alpha _{\infty }^2 \,({{\bar{\lambda }}}_1^\alpha + {{\bar{\lambda }}}_2^\alpha + {{\bar{\lambda }}}_3^\alpha - 3). \end{aligned}$$

The shear modulus $\mu$ and the nonlinearity parameter $\alpha$ are determined by fitting the model to experimental data. The volumetric part is defined as

12 $$\begin{aligned} \psi _{\textrm{vol}} = \kappa \frac{1}{4} \,(J^2 -1 -2 \textrm{ln} J), \end{aligned}$$

where $\kappa$, the bulk modulus, is determined from the shear modulus and the Poisson’s ratio, $\nu$, through the relation

13 $$\begin{aligned} \kappa = \mu \,\frac{2(1+\nu )}{3(1-2\nu )}. \end{aligned}$$

Following (9) the equilibrium stress is calculated from

14 $$\begin{aligned} \varvec{\tau }^\textrm{eq} = 2\,\dfrac{\partial \psi ^\textrm{eq}}{\partial {\textbf{b}}} \cdot {\textbf{b}} = \sum _{a=1}^{3}\dfrac{\partial \psi ^\textrm{eq}}{\partial \lambda _{a}}\lambda _{a}\;{\textbf{n}}_{a} \otimes {\textbf{n}}_{a}\,. \end{aligned}$$

To determine the Kirchhoff stress for each viscoelastic mode, $\varvec{\tau }_i$, the same Ogden type strain energy function is adopted with a similar split into an isochoric and volumetric part as in (10). The isochoric part is now given in terms of the isochoric elastic principal stretches ${\tilde{\lambda }}^e_i = (J^e_i)^{-1/3} \lambda ^e_i$ and is given by,

15 $$\begin{aligned} \psi _{i\,\mathrm {(iso)}} = 2\,\mu _i/\alpha _i^2 \, [({\tilde{\lambda }}^e_{i\,1})^{\alpha _i} + ({\tilde{\lambda }}^e_{i\,2})^{\alpha _i} + ({\tilde{\lambda }}^e_{i\,3})^{\alpha _i} - 3], \end{aligned}$$

and the volumetric part follows from (12),

16 $$\begin{aligned} \psi _{i\,\mathrm {(vol)}} = \kappa _{i} \frac{1}{4} \,(J^2 -1 -2 \textrm{ln} J), \end{aligned}$$

The Kirchhoff stress for each viscoelastic mode is expressed analogously to 14 as,

17 $$\begin{aligned} \varvec{\tau }_i= & {} 2\,\dfrac{\partial \psi _i}{\partial {\textbf{b}}_i^\textrm{e}} \cdot {\textbf{b}}_i^\textrm{e} \nonumber \\= & {} \sum _{a=1}^{3}\dfrac{\partial \psi _i}{\partial \lambda _{i\,a}^ \textrm{e}}\lambda _{i\,a}^\textrm{e}\;{\textbf{n}}_{i\,a}^\textrm{e} \otimes {\textbf{n}}_{i\,a}^\textrm{e} \nonumber \\= & {} \sum _{a=1}^{3}\tau _{i\,a}\;{\textbf{n}}_{i\,a}^\textrm{e} \otimes {\textbf{n}}_{i\,a}^\textrm{e}\,. \end{aligned}$$

It remains to specify the temporal evolution of the viscoelastic kinematics. To satisfy the reduced dissipation inequality for each mode (Govindjee and Reese 1997; Budday et al. 2017c), we choose the following evolution equation for the internal variable ${\textbf{b}}_i^{\textrm{e}}$,

18 $$\begin{aligned} -\,\mathscr {L}_{v}\,{\textbf{b}}_i^{\textrm{e}} \cdot ({\textbf{b}}_i^{\textrm{e}})^{-1} = \dfrac{1}{2\eta _i}\varvec{\tau }_{i}\,. \end{aligned}$$

The update of the non-equilibrium part of the constitutive equation in time is performed using an implicit time integration with exponential update, of which details can be found in Budday et al. (2017b).

### FE model

#### FE mesh generated from segmented brain image

**Fig. 1:**
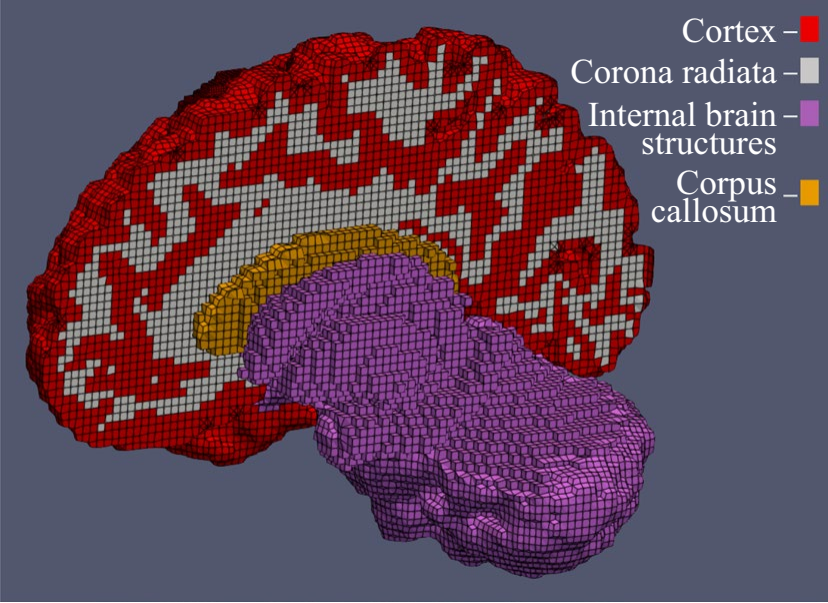
Segmentation of the three-dimensional brain consisting of four regions

A three-dimensional brain model that captures the major sulci and gyri was created from magnetic resonance imaging (MRI) data of a woman’s brain at the age of 30. FreeSurfer image analysis suite^1^ was used to segment the MRI images and MATLAB R2021b (MathWorks Inc, US) was used to clean the resulting voxel image, convert it into a mesh of hexahedral elements and apply smoothing to this mesh. Further details on its creation can be found in Griffiths et al. (2023). Figure 1 shows the segmentation of this model into 4 regions: cortex, corona radiata, corpus callosum and the remaining internal structures consisting of the amygdala, basal ganglia, brain stem, cerebellum, hippocampus and midbrain.

#### Material parameters

A compressible, viscoelastic material model is considered suitable to model neurosurgical procedures with sufficient accuracy (Kyriacou et al. 2002; Budday et al. 2017b). Using the material model described in Sect. 2.1 and based on the previous work of Budday et al. (2015, 2017b), we use two viscoelastic elements ($m=2$) to capture the viscoelastic response of brain tissue. We use the constitutive material parameters identified by Budday et al. (2017b) for conditioned brain tissue in four brain regions (shown in Table 1). A Poisson’s ratio of 0.49 is used throughout.

**Table 1:** Constitutive properties identified by Budday et al. (2017b) for the conditioned viscoelastic material of the four brain regions

	Ogden		$1{\textrm{st}}$ Maxwell element			$2{\textrm{nd}}$ Maxwell element		
	$\mu _{\infty }$	$\alpha _{\infty }$	$\mu _{1}$	$\alpha _{1}$	$\eta _{1}$	$\mu _{2}$	$\alpha _{2}$	$\eta _{2}$
	[kPa]	[–]	[kPa]	[–]	[kPa·s]	[kPa]	[–]	[kPa·s]
Cortex	0.42	$-$21.27	1.40	$-$14.66	3.05	0.56	$-$23.76	289.37
Corona radiata	0.16	$-$25.66	0.97	$-$25.35	2.19	0.25	$-$29.22	299.79
Internal structures	0.17	$-$21.52	0.68	$-$15.50	2.27	0.27	$-$22.76	240.17
Corpus callosum	0.04	$-$28.41	0.63	$-$27.01	1.62	0.16	$-$30.80	232.53

#### Boundary conditions

**Fig. 2:**
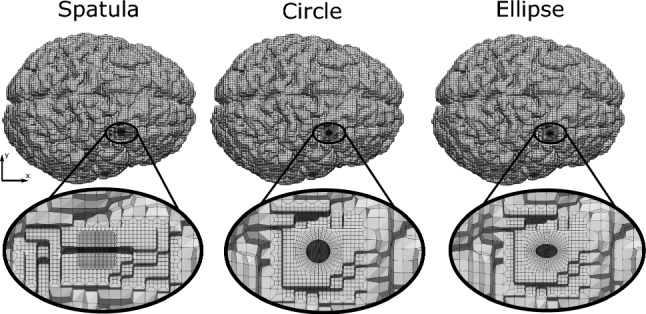
Magnified view of the initial mesh for each retraction mechanism (traditional spatula, tubular retractor with circular cross section, tubular retractor with an elliptical cross section), generated based on an octree mesh refinement is shown at the retraction location

Using the FE generated brain model from Sect. 2.2.1 and the hyper-viscoelastic material model described in Sect. 2.2.2, we aim to simulate a transsulcal brain retraction using three different mechanisms: two spatulas moving apart, an expanding circular retractor and an expanding elliptical retractor. The boundary conditions applied are taken from Awasthi et al. (2020), where the non-retracted hemisphere and the brain stem are fixed while the retracted hemisphere remains traction free. To improve the quality of the results and allow for a smooth transition between the element sizes at the retraction sites, an octree mesh refinement was applied (Schneiders 1998). This refinement method subdivides a hexahedral element into eight hexahedral elements. Elements of varying size are created in the transition zone between the refined and non-refined region. Fig. 2 shows a magnified view of the refined initial mesh setup for each retraction method. A summary of a mesh convergence study at the retraction site can be found in Appendix A.

**Fig. 3:**
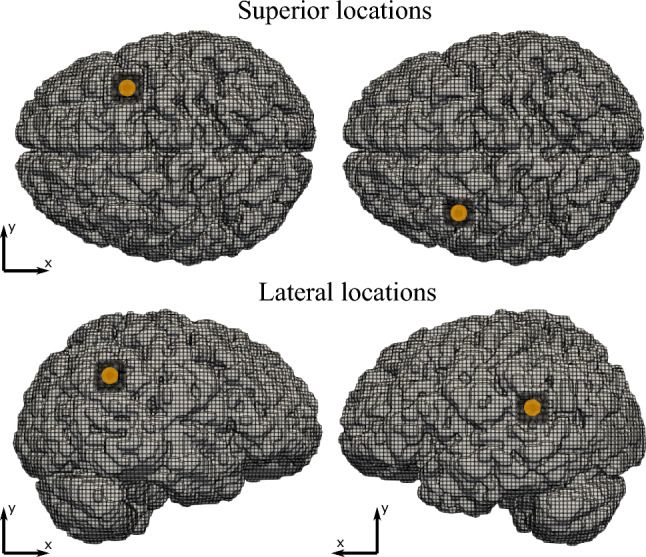
The four locations (two from top and two lateral) at which retraction is applied to the brain model. The retraction location is indicated on the brain with a yellow circle

In order to investigate the effects of location on the surrounding brain structures we chose four retraction locations, as shown in Fig. 3.

For the spatula retraction, an initial slit of 12 mm length and 1 mm gap was created to simulate the initial incision. This incision was made to a depth of 32 mm. The retraction of two spatulas of 4 mm width at a depth of 30 mm was simulated by prescribing the displacement of the nodes associated with these dimensions in the x- and y-direction. For the tubular retractions, an initial circular or elliptical puncture of 3 mm diameter was created in the brain mesh. The puncture replicates the catheter needle used to guide the retractors. This incision was made to a depth of 40 mm to account for a 30-mm tubular retractor and a 10-mm conical introducer located at the end of the retractors. The retraction was simulated by prescribing the radial displacement of nodes on the punctured surface to a depth of 30 mm.

The circular tubular retractors were expanded to a width of 10 mm and the elliptical retractor to a final major diameter of 10 mm and minor diameter of 6.67mm. Unlike tubular retractors, the retraction displacement of spatulas is not fixed. This displacement can vary between surgeries and surgeons but is most often larger than that for a tubular retractor (Evins 2017). In our simulations, the final spatula retractor displacement was set to 12.5mm. All simulations were performed at a retraction rate of 5 mm/min Budday et al. (2017c), Awasthi et al. (2020). After the final retraction displacement was reached the brain tissue was held at this displacement for 30 min to simulate the length of a typical neurosurgical procedure (Zhong et al. 2003; Awasthi et al. 2020).

All simulations were performed using the open source FE library deal.II^2^ previously implemented by Kaessmair et al. (2021).^3^

## Results and discussion

### Comparison of retraction mechanisms

**Fig. 4:**
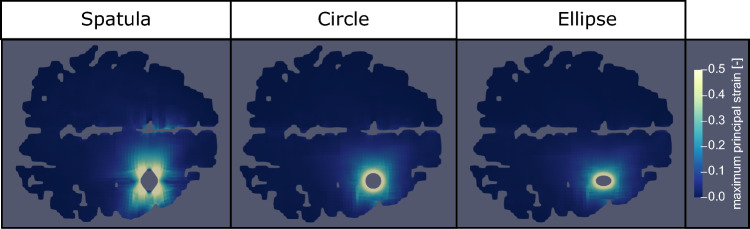
Simulated maximum principal strain distribution for a slice of the brain 2 cm below the surface of retraction for each retraction mechanism, using a traditional spatula, a tubular retractor with circular cross section, and a tubular retractor with an elliptical cross section. A strain limit of 50% has been set as a damage threshold based on the results of Franceschini et al. (2006)

To compare the three different retraction mechanisms, Fig. 4 shows the predicted maximum principal strain distribution on a transverse slice of the brain taken 2 cm below the surface. A strain limit of 50% has been set as a damage threshold. This value is based on the experimental results of Franceschini et al. (2006).

All mechanisms show strains above the damage threshold in the area immediately adjacent to the retraction. However, the simulated brain retracted using a spatula shows a much larger area of damage compared to either tubular retractor. A noticeable strain can even be noted on the non-retracted hemisphere. In comparison, the two tubular retractors show a regular and contained strain distribution with no visible strain on the non-retracted hemisphere.

**Fig. 5:**
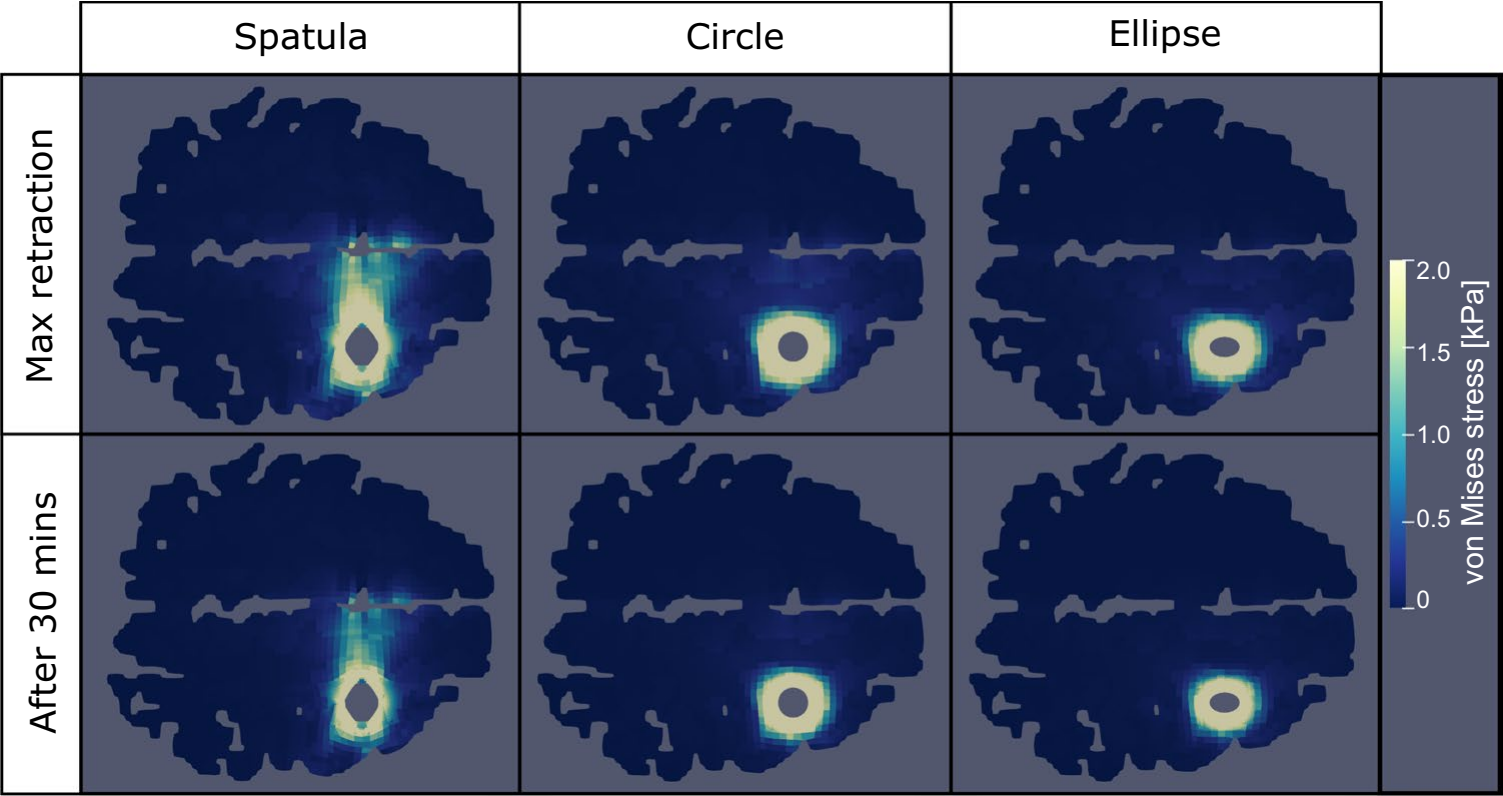
Simulated von Mises stress for a slice of the brain 2 cm below the surface of retraction for each retraction mechanism, using a traditional spatula, a tubular retractor with circular cross section, and a tubular retractor with an elliptical cross section. Two time points are shown: the first moment when the maximum retraction displacement has been reached and 30 min later whilst holding the maximum retraction displacement

Figure 5 shows the corresponding simulated von Mises stress distribution in the same location as in Fig. 4 at two time points: the first moment when the maximum retraction displacement has been reached and 30 min later whilst holding the maximum retraction displacement.

In all three models, a relaxation of the stress can be seen between the two time points, especially in regions further away from the retraction site. The relaxation observed for the spatula is much greater than for either tubular mechanism. The spatulas enforce the displacement over a relatively small area along the initial incision based on the width of the spatula. The tubular retractors enforce the displacement along the entire area of puncture. Since there is a larger area that is unprescribed in the spatula simulations, there is more freedom in the model to relax, thus greater stress relaxation can occur.

**Fig. 6:**
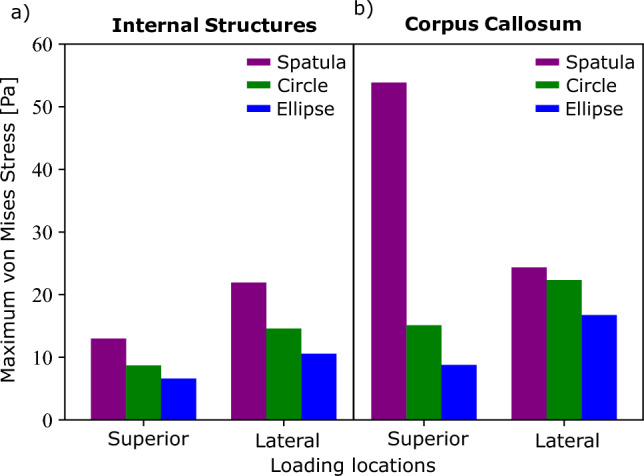
Maximum averaged simulated von Mises stress in two regions, internal brain structures and the corpus callosum, for three retraction mechanisms, a traditional spatula, a tubular retractor with circular cross section, and a tubular retractor with an elliptical cross section. The results are shown for two retraction locations: one lateral and one loaded from top

**Fig. 7:**
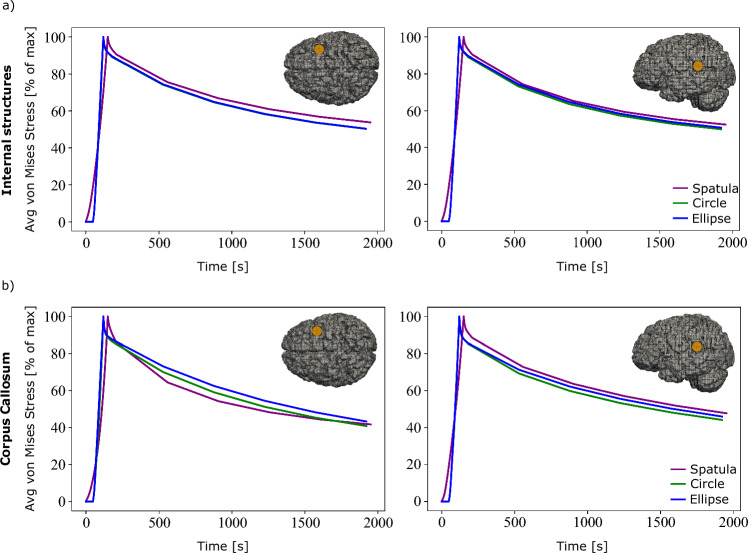
Normalized simulated von Mises stress over time in two regions, internal brain structures and the corpus callosum, for three retraction mechanisms, a traditional spatula, a tubular retractor with circular cross section, and a tubular retractor with an elliptical cross section. The results are shown for two retraction locations and are indicated on the brain inserts with a yellow circle

Similar to the strain in Fig. 4, the stress distribution resulting from using a spatula is both greater in area and much less regular than that of the tubular mechanisms. Significantly larger stress values are even noted in the non-retracted hemisphere. As one would assume, the areas of high stresses are areas, where secondary brain damage is more likely to occur. Those are smaller for the tubular retractors, which could motivate their preferred use over spatulas. There is a large amount of variability available to a surgeon when using a spatula, such as their position and width. The tubular retractors present almost no variability: the diameter is chosen beforehand and they provide a relatively large visually unobstructed surgical corridor through which the surgeon can work (Zagzoog and Reddy 2020). Therefore, the location and quantity of the high stress areas within the brain are easier to predict. The variability of spatulas makes predictions in these cases less reliable. The regularity of both the stress and strain distributions of the tubular mechanisms allows for a better prediction of which regions could be affected by retraction at difference sites when conducting surgical planning. We now look into the effects of each retraction mechanism on particular areas of the brain that are not in direct contact with the surgical instrument. Figures 6 and 7 show the maximum averaged predicted von Mises stress and the average predicted von Mises stress over time using each mechanism for (a) the internal structures of the brain and (b) the corpus callosum (the softest region of the brain (Budday et al. 2017b)). The results are shown for one location loaded from top and one lateral retraction location.

In Fig. 6, the spatula shows the largest produced maximum von Mises stress in the internal structures and the corpus callosum at both loading locations. The internal brain structures experience a 30–50% lower von Mises stress when the tubular retractors are used compared to the spatulas. In the corpus callosum, a 70% lower peak average stress is shown when loaded from the top using a tubular retractor compared to a spatula. For the lateral loading, the difference between the mechanisms is considerably smaller. The lowest average peak stress in all locations and brain regions is experienced when using an elliptical tubular retractor. This is expected due to the smaller minor diameter and thus the less overall retraction. While the elliptical cross section clearly reduces the area of visibility in retraction, the reduced risk of secondary brain damage to surrounding areas potentially prevails.

Figure 7a shows a similar stress relaxation profile in the internal structures at both locations. In the corpus callosum, a slightly larger amount of stress relaxation occurs when using a spatula compared to the other mechanisms when loaded from the top. This is consistent with the results shown in Fig. 5, where the spatula led to the most pronounced stress relaxation, particularly in areas far from the retraction site. For the laterally loaded condition, the corpus callosum shows slightly greater relaxation when using the circular or elliptical retractor.

An important note to make on the different retraction mechanisms is the variability that is allowed with the spatula. The spatula mechanism can vary in width, placement, stability, retraction displacement and number of spatulas used. These factors can be both beneficial and harmful to the surrounding tissue. For example, the use of intermittent and multi-spatula reaction has been shown to produce lower reaction forces on the brain surface (Andrews and Bringas 1993; Awasthi et al. 2020), but excessive retraction of the brain or accidental slipping of the instrumentation may cause unexpectedly high forces. On the other hand, tubular retractors have very little variability: the diameter is chosen beforehand and they provide a relatively large visually unobstructed surgical corridor through which the surgeon can work (Zagzoog and Reddy 2020). Tubular retractors can thus be considered a more consistent and predictable mechanism. This predictability allows for greater accuracy in the planning of complex and dangerous neurosurgical procedures but also hinders potentially necessary variability to handle unforeseen situations.

This is the first FE study where the effects of different types of retraction mechanism on brain tissue loadings have been considered. From our study, the tubular retractors predict lower maximum von Mises stresses when compared to spatulas. The tubular retractors also showed smaller and more confined areas above current known damage thresholds close to the retraction site. This supports the clinical observations that tubular retractors could be safer than spatulas in certain brain surgeries scenarios.

### Effects of location changes

Oftentimes, during surgical planning different locations are considered and the decision for the best location is made based on the location of the entity to be accessed, the nearby brain structures, and the distribution of the white matter tracts (Hendricks and Cohen-Gadol 2016). We explore the effects of location changes on the predicted von Mises stress of the internal structures of the brain and the corpus callosum.

**Fig. 8:**
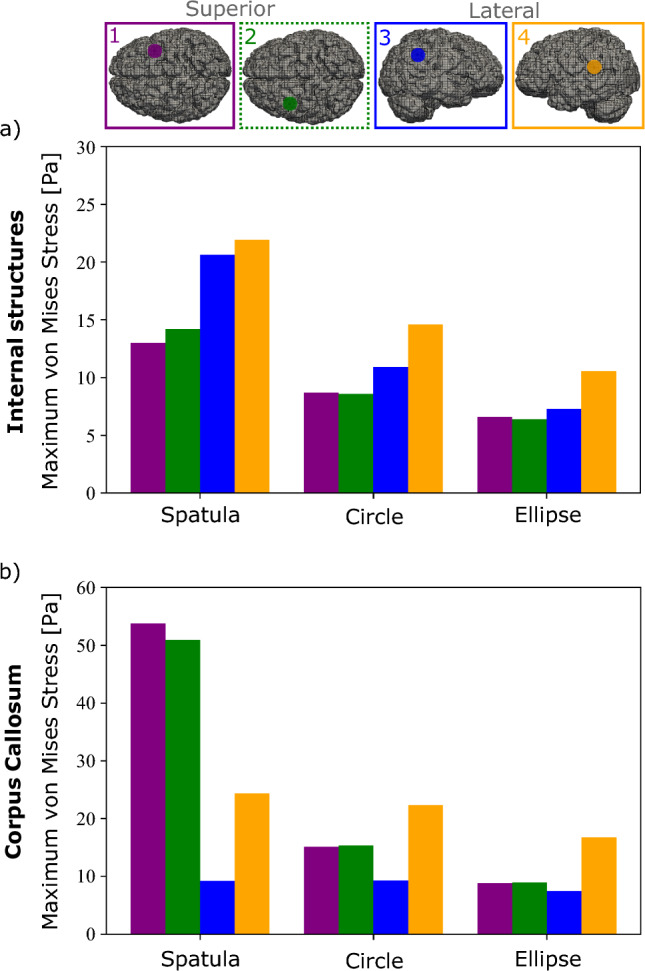
Maximum average simulated von Mises stress in the **a** internal brain structures and **b** corpus callosum for four different locations and each of the three retraction mechanisms. The retraction locations are indicated on top

Figure 8 shows the maximum averaged von Mises stresses for the internal structures and corpus callosum at the four locations for each mechanism.

In Fig. 8a, the von Mises stress is higher for the laterally loaded locations compared to the locations loaded from the top for all mechanisms. Additionally, little difference is seen between the two locations loaded from the top in either region. Utilizing a spatula shows a larger von Mises stress when loaded laterally. The tubular retractors have similar maximum von Mises stresses when loaded from the top but differing values when loaded laterally. Location three shows lower stress values, similar to the ones loaded from top, especially when using an elliptical tube. Location four has the greatest peak von Mises stress when using any mechanism. We attribute this to location four’s proximity to the area. Location four is positioned directly above the internal brain structures, while location three is slightly further away. It should be noted, however, that the maximum von Mises stress is higher for the spatulas than for the tubular retractors at all locations.

The low stiffness of the corpus callosum causes it to be much more sensitive to the location selection, as shown in Fig. 8b. Using the spatula, both lateral locations led to lower average stress values compared to the locations from the top. In the corpus callosum, an 80% difference in average stress is noted when comparing location one and location three. The location sensitivity of stresses in the corpus callosum can also be seen with regard to the tubular retractors. Similar to the results shown in Fig. 8a, lateral location four shows higher average stresses than lateral location three. However, this is shown to a greater degree here with location three causing the lowest average stress out of all four locations, and location four causes the highest average stress.

**Fig. 9:**
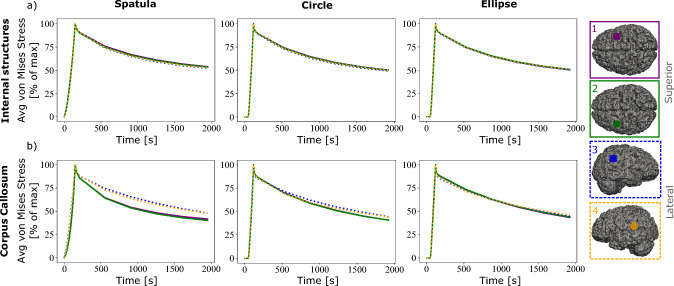
Normalized average simulated von Mises stress over time in the **a** internal brain structures and **b** corpus callosum for four different locations and each of the three retraction mechanisms. The retraction locations are indicated on the right hand side

Figure 9 shows a comparison of the predicted average stress values over time at the four different locations (shown on the right hand side of Fig. 9) for each mechanism.

Figure 9a shows a similar stress relaxation response for the internal structures for all three mechanisms at all four locations. Figure 9b shows a more pronounced stress relaxation within the region of the corpus callosum for the loadings from the top when using a spatula. This, however, is much less pronounced when using the tubular retractors.

This FE study is the first to consider different surgical sites for retraction in a human brain. From these results, we can see how the combined location and mechanism affect the predicted von Mises stresses in different areas of the brain, even far from the retraction site. It is not possible, however, to conclude that one specific retraction device is superior over the other when considering secondary damage. Figure 8 shows that a spatula lead to lower stresses in location one compared to a circular retractor in location three. This complex interaction is not fully understood yet. But the effects of location are clearly shown here to be an important parameter to consider for retraction brain surgery in the future.

### Effects of incorporating regional heterogeneity

The brain consists of several anatomical regions that have varying functional demands and also differ in their mechanical properties (Budday et al. 2017b; Hinrichsen et al. 2023). To show the necessity of taking these variations into account in a full three-dimensional FE model of the brain, we compare the model with four distinct regions (4R: cortex, corona radiata, corpus callosum, and internal brain structures) to a homogeneous (1R) model, whereby the parameters were calculated from the volume average of the four regions.

The material parameters of the homogeneous model are given in Table 2. Additionally, the percentage difference of these parameters from their region-specific values (given in Table 1) is provided. In the homogeneous model, the hyperelastic and viscoelastic values differ significantly from their region-specific values. The corpus callosum is modelled to be much stiffer in the 1R model than it is in the 4R model and the viscosity of this region is also highly overestimated by the 1R model. The cortex is the only region that is modelled to be slightly softer than in the region-dependent model.

**Table 2:**
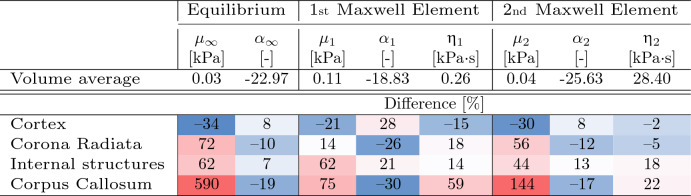
Volume averaged material parameters used in the homogeneous (1R) model and the percentage difference of these material parameter from those used in the region-specific (4R) model accounting for the distinct mechanical properties of the cortex, corona radiata, corpus callosum, and internal brain structures

Table 3 summarizes the volume average time constants $(\tau _{i}= \eta _{i}/\mu _{i})$ calculated from the volume average viscoelastic material parameters and compares them with the region-specific values. The time constant for the first viscoelastic element $\tau _1$ only shows a large difference for the internal structures. The time constant for the second viscoelastic element $\tau _2$ shows more deviation in all regions. The greatest difference appears for the corpus callosum.

**Table 3:**
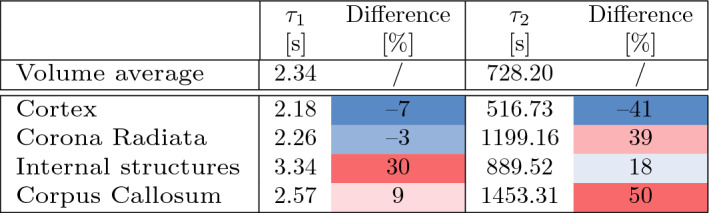
Characteristic time constants $(\tau _{i}= \eta _{i}/\mu _{i})$ near thermodynamic equilibrium calculated from the volume average material parameters and the percentage difference from the time constants calculated for the region-specific (4R) model accounting for the distinct mechanical properties of the cortex, corona radiata, corpus callosum, and internal brain structures

**Fig. 10:**
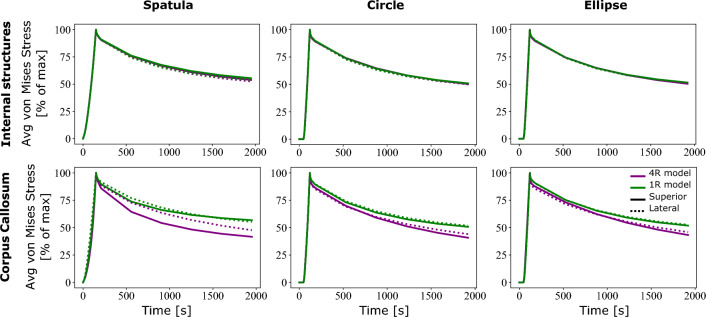
Normalized average simulated von Mises stress over time in the four segmented regions of the brain for each of the three retraction mechanisms comparing two different models: A fully homogeneous model (1R) and the region-dependent model (4R). Each retraction was performed at two locations, one from the top and one lateral

**Fig. 11:**
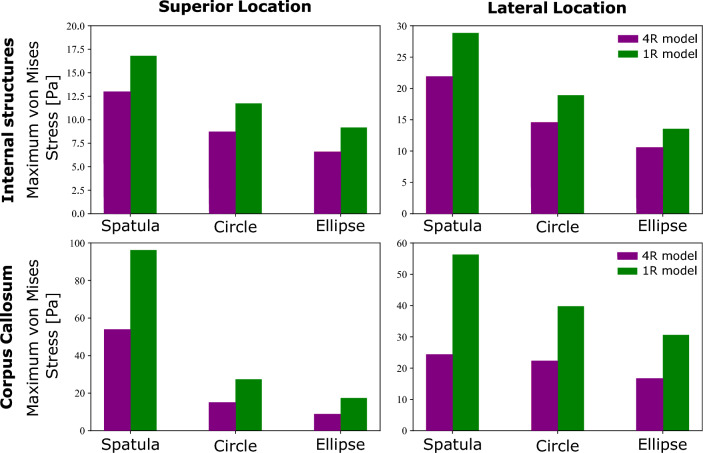
Maximum averaged simulated von Mises stress in the regions of the internal structures and corpus callosum of the brain for each of the three retraction mechanisms comparing two different models: A fully homogeneous model (1R) and the region-dependent model (4R). Each retraction was performed at two locations, one from the top and one lateral

Upon investigation of the regional responses, the cortex and corona radiata did not show significantly different responses. This may be due to the larger area of constraint on this model on those areas and the larger areas of prescribed displacement of these regions. For this reason, these regions are not considered for comparison. Fig. 10 shows the normalized average von Mises stress over time in the internal structures and corpus callosum between the 4R and the 1R model. Figure 11 shows a comparison of the maximum average von Mises stress for these two regions.

There is no visible difference in the stress relaxation in the internal structures, in spite of large difference between the volume averaged and regionally specific time constants. Slight differences can be noted in the relaxation behaviour of the corpus callosum, where the 4R model shows a more pronounced stress relaxation for all three mechanisms. Differences, however, can be noted in the predicted maximum von Mises stress in Fig. 11.

In internal structures, differences of between 20 and 30% are seen across all three mechanisms. Within the corpus callosum, even larger differences between 45 and 60% are noted, as expected from the large difference in the parameter values reported in Table 2.

**Fig. 12:**
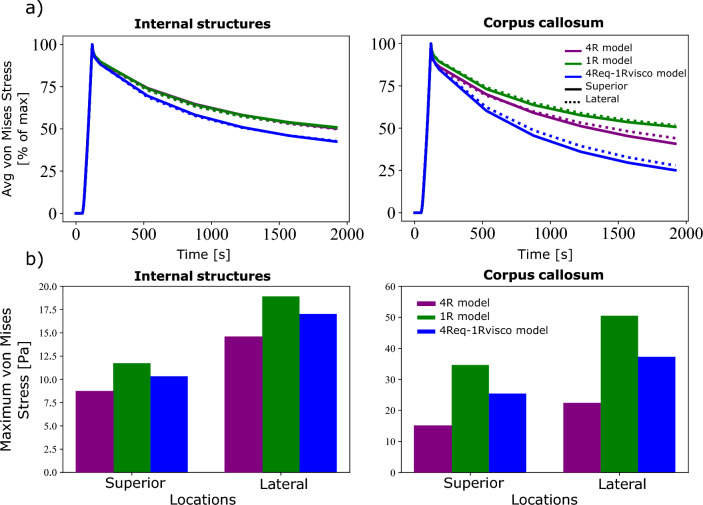
**a** Normalized average and **b** maximum average simulated von Mises stress in the internal structures and corpus callosum for a circular retraction mechanism comparing three different models: A fully homogeneous model (1R), a heterogeneous hyperelastic but homogeneous viscoelastic model (4Req-1Rvisco) and the fully heterogeneous model (4R). Each retraction was performed at two locations, one from the top and one lateral

Figure 11 clearly shows that region-specific parameters are required for simulations of brain surgery, especially in the region of the corpus callosum. However, interestingly, while we found differences between the 1R and the 4R model in terms of the peak stress values, little difference is shown in their stress relaxation behaviour in Fig. 10—albeit the significantly different time constants. In order to explore the effects of heterogeneous viscoelastic properties in more detail, we performed simulations of the fully segmented brain with regionally heterogeneous hyperelastic properties but homogeneous viscoelastic properties (4Req-1Rvisco) undergoing a circular tube retraction. These were then compared to both the fully heterogeneous (4R) model and the fully homogeneous (1R) model. The maximum average von Mises stress and average von Mises stress over time are shown in Fig. 12.

In this figure, the effects of the higher time constants of the homogeneous model can be seen. A more pronounced stress relaxation behaviour (compared to Fig. 10) can be seen in the internal brain structures. The corpus callosum the 4Req-1Rvisco model also undergoes a greater stress relaxation. It is thus not appropriate to model the viscoelastic behaviour of these regions as a homogeneous material.

For the internal brain structures, the 4Req-1Rvisco model shows a lower maximum stress value than the 1R model, but it is still greater than the 4R models. A similar trend is seen for the corpus callosum. This indicates that both the equilibrium response and the viscoelastic response of the material affect the peak von Mises stress predicted by the simulations.

Comparing Figs. 11 and 10 with 12, we observe that it is important to incorporate heterogeneous material parameters for both the hyperelastic and viscoelastic behaviour of the brain. While regions close to the site of loading show similar hyperelastic and viscoelastic responses independent of the model used, in regions far away, the average stress response differs greatly.

## Conclusions and recommendations

FE analysis presents a unique opportunity to model neurosurgical procedures under controlled conditions, whereby equipment can be tested and probed easily and efficiently. In this study, we have used FE analysis to provide insights into how three different retraction mechanisms affect the brain. We compared traditional spatulas to tubular retractors and found higher predicted stresses in the brain when using traditional spatulas in several different locations. We also showed how changes in the location of insertion can greatly affect the predicted stress results. By using FE analysis, different locations of access could thus be probed to find those that minimize the expected stresses throughout the brain. While we had to make certain assumptions to model this neurological procedure, we strongly believe that our analyses have provided valuable insights into how different retraction mechanisms affect the surrounding brain tissue.

This study supports the clinical observations that tubular retractors may cause less secondary brain damage than traditional spatulas. Our simulations show that, at the same location, the tubular retractors predict a lower maximum von Mises stress in the internal structures of the brain and in corpus callosum. As there are large variations inherent in these types of surgeries, one cannot, however, assume that the tubular retractor is always the safer option. For example, when retraction location was varied, certain locations showed a lower predicted maximum stress using spatulas compared to using tubular retractors at other locations. This shows an important interplay between these two parameters that should be considered before surgery. By using FE analysis these parameters (and other variations such as cutting depth and patient-specific brain anatomy) are easier and safer to investigate.

We have also investigated the importance of using region-specific hyperelastic and viscoelastic material properties and revealed interesting results for the modelling of three-dimensional brains during surgery. Our study highlights that both region-dependent hyper- and viscoelastic parameters should be used as they greatly affect the predicted stress in the internal structures of the brain and the corpus callosum.

In conclusion, our study highlights the relevance of simulating neurosurgical procedures and the importance of region-specific parameters for clinically relevant predictions. Our analysis presents a preliminary example of how FE simulations could be used to find the safest and most suitable route to perform retraction based on models that are patient-specific, both anatomically and mechanically.

## Figures and Tables

**Fig. 13:**
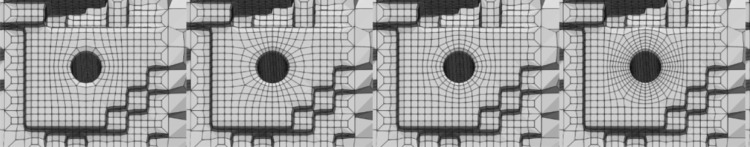
Four meshes at the circular retraction site of decreasing element size to be used to conduct the mesh convergence study

**Fig. 14:**
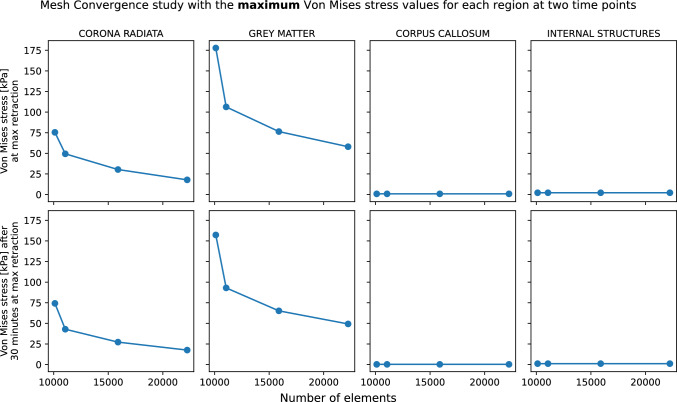
The maximum von Mises stress predicted using the different meshes introduced in Fig. 13 in each region of the segmented brain model

## Appendix A: Mesh convergence study

We have conducted a convergence study for various element sizes at the mesh refinement site. The four meshes of decreasing element size and corresponding results are shown in Fig. 13.

Figure 14 shows a clear path towards mesh convergence in the regions of the corona radiata and the grey matter. Due to a restriction to hexahedral elements in deal.II and the complex nature of the brain model, we are unable to refine the mesh any further. However, by extrapolation of the curves, we predict convergence to be achieved with only one more step. As we do not compare the maximum predicted von Mises stress for the corona radiata and the grey matter when comparing the mechanisms in Fig. 6 or for the location comparisons in Fig. 9, we believe this final mesh to be sufficient. For these figures, we limited ourselves to the corpus callosum and internal structures, which show a convergence in the mesh convergence study.

Using the element size of this final converged mesh, we refined the initial meshes of the other two retraction mechanisms with similarly sized elements.

## Footnotes

1 https://surfer.nmr.mgh.harvard.edu/

2 https://www.dealii.org/

3 The code can be found at https://github.com/BRAINIACS-Group/efiSim1F_Brain_Retraction.
